# Aldolase predicts subsequent myopathy occurrence in systemic sclerosis

**DOI:** 10.1186/ar3888

**Published:** 2012-06-22

**Authors:** Cécile Tolédano, Murielle Gain, Adrien Kettaneh, Bruno Baudin, Catherine Johanet, Patrick Chérin, Sébastien Rivière, Jean Cabane, Kiet Phong Tiev

**Affiliations:** 1 University Paris VI, AP-HP, Saint Antoine Hospital, 184 rue du Faubourg Saint-Antoine, 75571 Paris Cedex 12, France; 2 Department of Internal Medicine, Biochemistry Laboratory, 184 rue du Faubourg Saint-Antoine, 75571 Paris Cedex 12, France; 3 Immunology Laboratory, 184 rue du Faubourg Saint-Antoine, 75571 Paris Cedex 12, France

## Abstract

**Introduction:**

Myopathy related to systemic sclerosis (Myo-SSc) is a disabling and unpredictable complication of SSc. We assessed the predictive value of serum aldolase, creatine kinase (CK), alanine transaminase (ALT), aspartate transaminase (AST) and C-reactive protein (CRP) to estimate the risk of developing Myo-SSc.

**Methods:**

We enrolled 137 SSc patients without proximal muscle weakness in a prospective monocentric study to follow them longitudinally over a four-year period. The risk of occurrence of Myo-SSc was ascertained according to the European NeuroMuscular Centre criteria and was analyzed according to levels of plasma aldolase, CK, transaminase enzymes and CRP at inclusion. Performance of each parameter to predict Myo-SSc occurrence was assessed and compared with the others.

**Results:**

The area under the receiver operating characteristic curves (ROC) of plasma aldolase for Myo-SSc occurrence prediction was 0.80 (95% CI: 0.67 to 0.94, *P *< 0.001), which was higher than that of plasma CK (0.75, *P *= 0.01), and that of ALT (0.63, *P *= 0.04). AST and CRP had no predictive value for Myo-SSc occurrence. The best cut-off of aldolase for prediction of Myo-SSc occurrence within three years after inclusion was 9 U/L and higher than the upper normality limit (7 U/L), unlike that of CK and ALT. Myo-SSc occurred more frequently in patients whose plasma aldolase was higher than 9 U/L. Adjusted Hazard Ratio for patients with aldolase > 9 U/L was 10.3 (95% CI: 2.3 to 45.5), *P *< 0.001.

**Conclusions:**

Increased plasma aldolase level accurately identified SSc patients with high risk to develop subsequent Myo-SSc. This could help initiate appropriate treatment when the disabling muscle damage is still in a reversible stage.

## Introduction

Systemic sclerosis (SSc) is a connective tissue disease characterized by microvasculature damages, immune system dysfunction and excessive deposition of collagen in skin and other organs [1]. The prevalence of muscle involvement in SSc previously reported ranges widely from 16% to 81% according to different diagnosis criteria used for muscle involvement [2-4]. Proximal muscle strength weakness is a hallmark of a severe form of myopathy related to SSc (Myo-SSc) having a tangible impact on the impairment of quality of life. Current investigations focused on the diagnosis and therapy in Myo-SSc, and copy exactly those from idiopathic inflammatory myopathies (IIM) [5]. However, the pathology pattern of muscle in SSc is heterogeneous (from myositis to muscle fibrosis) and different from those of IIM [3,6]. Available therapies, including corticosteroids, immunosuppressive agents and intravenous immunoglobulin, may slow down the muscle inflammation progression to prevent muscle strength deterioration [7,8], but early diagnosis is important to avert irreparable muscle damages and progression of myositis that leads to bedridden patients with Myo-SSc becoming unresponsive to appropriate therapy. Thus, there is a need to develop biomarkers that could permit early identification of SSc patients who are in danger of having subsequent Myo-SSc. The goal is to provide them with appropriate therapy on time.

A partially validated laboratory test to assess the activity of myopathies is to assay the muscle enzymes: creatine kinase (CK), transaminase enzymes including aspartate transaminase (AST), and alanine transaminase (ALT) and aldolase A, an isomer present in muscles, other isomers (aldolase B and C) are present in brain or liver and are not found in plasma, except in liver diseases. Elevated serum or plasma levels of CK, transaminase enzymes or aldolase A were observed in patients with SSc with neither muscle weakness nor having engaged in any physical activities the days prior to the muscular enzymes assay. This suggests that isolated increments may be associated with underlying extended muscle damage and may reflect the early stage of this muscle disease [9]. On the other hand, in Myo-SSc, inflammatory cells and activated auto-reactive T cells contribute partly to muscle damage. Increased inflammatory markers, such as C-reactive protein (CRP), might also indirectly reflect inflammatory muscle injury and could be a potential predictive marker of subsequent Myo-SSc occurrence.

Taken together, we hypothesized that muscle enzymes, including aldolase A, CK, AST, ALT and CRP, could help identify patients who were going to have subsequent disabling Myo-SSc, but without proximal muscle weakness. Therefore, in this prospective cohort study, we assessed whether or not these biomarkers might estimate the risk of subsequent Myo-SSc.

## Materials and methods

### Study design

The inclusion period for this prospective monocentric cohort study was from November 2004 to January 2007 in the Department of Internal Medicine, Saint Antoine Hospital, University Paris VI, Paris, France, and the follow-up of this time-to-event driven study ended in April 2010. Patients who met the following criteria were eligible for the study.

### Inclusion criteria

Patients were considered eligible for inclusion if they were older than 18 years and had a diagnosis of SSc, according to the American College of Rheumatology criteria [10]. The subsets of disease were defined according to LeRoy's criteria [11]. Patients treated by immunosuppressive therapy were also eligible. More importantly, patients must not have had any proximal muscle weakness. Muscle strength was checked according to the eight manual muscle testing (8-MMT).

### Eight manual muscle testing

8-MMT was used to check the muscle strength according to the Medical Research Council scale [12,13] and Kendall's scale [14]. The intensity of the muscular deficit was determined from the total quotation of eight muscles: neck flexors, deltoid, biceps, psoas, maximus and medius gluteus, and quadriceps. A training of 8-MMT was performed before the beginning of the study. Pr Cherin (P) taught physicians and physiotherapists to score and to test muscle strength. To insure that proximal muscle involvement was symmetrical, we checked the muscle strength bilaterally. If muscle strength was not symmetrical, we used the most severe score of the two sides. The variations of muscle strength score between the two sides were less than 2%. The variation between two observers was less than 4% during the training period. The maximum score (normal muscle power) is 80 points, and represents the unilateral muscle strength score (70 points) plus the neck flexors strength (10 points) according to Kendall scoring [14]. The muscle strength was considered as normal if the 8-MMT score was > 72 points. A proximal muscle weakness was considered present, relevant and sustained if it was observed by the investigator and physiotherapist performing the 8-MMT with one month between the two testings. If there was disagreement of more than 5% of the 8-MMT score between the two tests at the same visit, a third confirming testing was performed. The mean value of the three 8-MMT was kept as the 8-MMT score of the visit. To exclude SSc patients with minor muscle involvement at the inclusion visit, only the patients with normal muscle strength (8-MMT score of > 72 points) were eligible for this cohort study. An 8-MMT score of less than 72 points represents a reduction of more than 10% of the normal muscle power (80 points).

### Exclusion criteria

Patients with proximal muscle weakness (8-MMT score ≤ 72) or known myositis were excluded. Those with the following conditions that may increase blood aldolase, AST, ALT, CK, and CRP were excluded: infection, hypothyroidism, hepatitis, chronic haemolysis, recent myocardial infarction (less than three months), or recent leg or arm trauma. Patients who were unable to perform correctly the initial muscle strength checking for any reason were also excluded.

### Data collection

The study was approved by the local ethics committee. After written informed consent, patients underwent a complete evaluation and the following data were collected: age, gender, subtype of SSc, disease duration (duration between the first non-Raynaud's symptom and date of enrolment).

### Blood sample

Blood tests including serological testing for antinuclear antibodies (ANA), antitopoisomerase I antibody (ATA), anti-centromere antibody (ACA), anti-ribonucleoprotein antibody (U1-RNP), and anti-polymyositis-scleroderma antibody (PM-Scl) were performed. ANA were detected by indirect immunofluorescence using slides of monolayer Hep-2 cells (Kallestad, BioRad, Marnes la Coquette, France) as the substrate. Seropositivity for ANA was defined as 1:80 or higher. Centromere protein B, antitopoisomerase I, U1-RNP, anti RNA polymerase III and PM-Scl were identified by immunodot (Euroimmun, BioAdvance, Bussy Saint-Martin, France). We used homemade immunodiffusion for Pm-Scl antibody, which detects both anti-PMScl-100 and anti-PMScl-75. In patients with myositis (*n *= 5), a discrepancy between immunodiffusion and immunodot methods (that is, immunodiffusion positive for anti Pm-Scl antibody and immunodot positive for anti Pm-Scl-100 KDa antibody), we have shown the presence of anti-PMScl-75 antibody by dot blot method (Euroline myositis-antigen profil 3, Euroimmun AG, Lubeck, Germany).

All sera were tested by homemade immunodiffusion (ID) which detect both anti-PMScl-100 and anti-PMScl-75, in case of discrepancy between immunodiffusion and immunodot methods, detection of anti-PMScl 100 and anti-PMScl-75 separately was performed by dot blot (Euroline myositis-antigen profil 3, Euroimmun AG, Lubeck, Germany).

### Aldolase, creatine kinase and C reactive protein measurements

Blood samples were collected by venipuncture on Li-heparinate 5 mL tubes from patients after 72 h of resting and an overnight fasting. Plasma aldolase and CK activities were performed by enzymatic methods at inclusion and at the diagnosis of myopathy. For aldolase determination, we adapted a kit from Randox on Synchron CX-4 (Beckman-Coulter, Brea, CA, USA) and using quality controls from Roche, (Burgess Hill, United Kingdom); this assay measures only aldolase A (named aldolase), but not the other isoforms, aldolases B and C. AST, ALT, CK activities and CRP were measured on Olympus AU-800 (Olympus) using commercial kits and both internal and external quality controls. The upper limits for plasma aldolase and CK were 7 U/L and 160 U/L, respectively, as stated by the kit manufacturers. The upper limit for CRP was 4 mg/L and 32 U/L for both transaminase enzymes (AST and) ALT in females, but 35 U/L for AST and 43 U/L for ALT in males.

### Follow-up

#### Endpoint: occurrence of myopathy related to scleroderma

The occurrence of Myo-SSc was defined as events that took place during the follow-up. Visits were scheduled every year over a four-year period and more frequently when clinically indicated. Weakness was defined by the loss of eight points, representing a relative decrease of 10% of the maximal score from baseline at the 8-MMT score. We chose this magnitude of change to detect significant muscle weakness requiring therapy. Changes in muscle strength were assessed by 8-MMT at each visit by the physician. If the proximal muscle weakness (8-MMT score < 72) was present, the physiotherapist tested the patient again within a month to insure the presence of sustained muscle weakness [15].

Patients with a relevant proximal muscle weakness underwent an electromyography (EMG) or/and a thigh muscle resonance magnetic imaging (RMI) and/or a muscle biopsy. The diagnosis of Myo-SSc was defined as the presence of muscle weakness and at least two of the three following criteria according to the 119^th ^European Neuromuscular Centre (ENMC) International Workshop criteria [5]:

1. Electromyographic triad of myopathy on EMG: low voltage and short duration potential, fibrillation or sharp wave.

2. Characteristic features of muscle involvement in thigh muscle RMI: hyperintense signal and enhancement on STIR and fat-saturated gadolinium enhanced T1 weight images.

3. Evidence of myositis in muscle biopsy specimen: myofibre atrophy and necrosis/regeneration; inflammation; fibrosis; microangiopathy; vasculitis involving small arteries; mitochondrial abnormalities and neuropathic changes.

On the other hand, patients with normal muscle strength were also followed up on a regular basis with one visit a year and more frequently if symptoms required. During the follow-up, patients were informed about their plasma aldolase levels and the need to contact us if they had a feeling of muscle weakness. The isolated increased aldolase level does not induce a change of medication or behavior in the treating physician. At the additional visit, we performed 8-MMT again. Only patients with reduction of muscle strength of more than 10% from the baseline score of 8-MMT required further investigations for myositis.

### Statistical analysis

All data are given as mean ± standard deviation (SD) and percentage for continuous variables and categorical variables respectively. A *P-*value < 0.05 was considered as statistically significant.

We used receiver operating characteristic curves (ROC) analysis to assess both the optimal threshold with the highest Youden index and the diagnostic performance of muscular enzymes, which identified SSc patients who were going to have subsequent disabling Myo-SSc within the three years after inclusion (positive if Myo-SSc occurred within three years of follow-up and negative if Myo-SSc did not occur).

Second, SSc patients were categorized into two groups: patients with muscular enzymes levels higher than the best threshold and the remaining patients. To estimate the predictive value of muscular enzymes on subsequent Myo-SSc occurrence during the whole follow-up, cumulative risks were computed using the Kaplan-Meier analysis. We used Cox proportional hazard model multivariate analysis to assess the risk of developing Myo-SSc for patients according to the plasma level of pertinent predictive biomarkers at inclusion. Odds-ratios for Myo-SSc occurrence according to pertinent predictive biomarkers were estimated by the Cox proportional hazard model.

For all parameters, the conformity with proportional hazards assumption was tested by linear regression of Schoenfeld residuals on the time variable in observations with event = 1. For categorical parameters, the conformity with proportional hazards assumption was verified graphically and, in addition, by Kaplan Meier curves construction. Only parameters fulfilling the proportional hazards assumption were used in the Cox analysis.

## Results

### Characteristics of population at baseline

**Figure 1:**
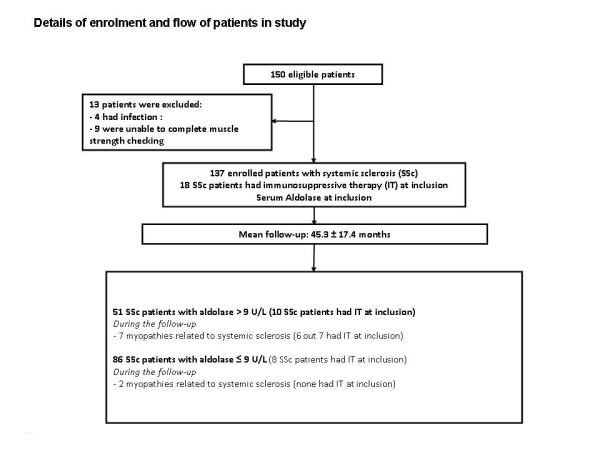
**Details of enrolment and flow of patients in the study**.

One hundred, fifty patients with SSc were eligible in this prospective study. Four SSc patients with systemic infection and nine other patients who were unable to complete muscle strength checking procedure were excluded. Among the nine SSc patients excluded for the presence of proximal muscle weakness at inclusion, the diagnosis Myo-SSc was made in four (Figure 1 illustrates the study design). We thus included 137 patients consecutively in the study (Table 1). The mean age was 54.8 ± 12.9 years. The mean disease duration was 12.2 ± 12.0 years. The rate of the diffuse form of disease was 31%. Fifty-six percent of patients experienced myalgia. Thirty-seven among 137 patients had ATA, 62 had ACA, 8 had U1-RNP antibody, 6 patients U3-RNP antibody, 5 patients anti-PM-Scl antibody and 4 anti-RNA polymerase III. Any discrepancy between immunodiffusion and immunodot methods for Pm-Scl antibody was detected. The mean level of CRP was 5.2 ± 7.6 mg/L. Mean levels of aldolase and CK were 10.3 ± 7.5 U/L and 111.1 ± 142.1 U/L, respectively. During a mean follow-up period of 45.3 ± 17.4 months, no patient was lost. Among 14 patients with SSc who experienced proximal muscle weakness during the follow-up, 5 had transient proximal muscle weakness and 9 had Myo-SSc according to the 119^th ^ENMC International Workshop criteria (Table 2) [5]. The 8-MMT score from five SSc patients fluctuated during the follow-up. We performed EMG and RMI in these five patients. EMG and RMI showed no abnormality consistent with a myositis. We retested these five patients in the subsequent scheduled visits. Their 8-MMT score became higher than 72 points.

**Table 1:** Characteristics of the patients at inclusion

	Patients with systemic sclerosis (*n *= 137)
**Age (years)**	54.8 ± 12.9
**Female, n (%)**	125 (91.2)
**Duration of SSc disease (years)**	12.2 ± 12.0
**Diffuse, n (%)**	42 (31)
**Modified Rodnan skin score**	8.1 ± 6.0
**Myalgia, n (%)**	77 (56)
**DLCO (% pred)**	64 ± 17
**ILD, n (%)**	51 (37.1)
**ATA, n (%)**	37 (27)
**ACA, n (%)**	62 (45)
**Anti U1-RNP antibody**	8 (5.8)
**Anti U3-RNP antibody**	6 (4.3)
**Anti PM-Scl antibody**	5 (3.6)
**Anti RNA polymerase III antibody**	4 (2.9)
**C-reactive protein (mg/L)**	5.2 ± 7.6
**Plasma aspartate transaminases (0 to 32 U/L)**	23.1 ± 9.3
**Plasma alanine transaminases (0 to 32 U/L)**	21.5 ± 14.0
**Plasma aldolase (0 to 7 U/L)**	10.3 ± 7.5
**Plasma creatine kinase (0 to 160 U/L)**	111.1 ± 142.1
**Immunosuppressive treatment, n (%)**	18 (13)

% of pred, percentage of predicted value; ACA, Anti-centromere antibodiy; U3-RNP, Anti U3-RNP antibody; anti-PM-Scl, anti-polymyositis-scleroderma antibody; ATA, Anti-topoisomerase I antibody; DLCO, diffusing capacity of carbon monoxide; ILD, interstitial lung disease; PM-Scl, polymyositis scleroderma; sPAP, Systolic pulmonary artery pressure assessed by echocardiogram; U1-RNP, U1-ribonucleic protein. Among 18 patients with immunosuppressive therapy, nine had corticosteroid dose less than 10 mg/day.

**Table 2:** Clinical features at diagnosis of patients with myopathy related to systemic sclerosis

N°	Type	Antibody	At baseline				At diagnosis							
			Aldolase (0 to 7 U/L)	AST (0 to 32 U/L)	ALT (0 to 32 U/L)	CK (0 to 160 U/L)	8MMT (0 to 80)	Aldolase (0 to 7 U/L)	AST (0 to 32 U/L)	ALT (0 to 32 U/L)	CK (0 to 160 U/L)	EMG	Thigh muscle RMI	Muscular biopsy
1	dSSc	ATA	16	38	12	34	66	19	172	49	800	+	-	+
2	dSSc	ATA	9	35	20	11	60	9.7	58	28	234	+	-	+
3	lSSc	ACA	15.8	36	24	25	64	40	86	32	100	-	+	+
4	lSSc	ATA	23.5	25	31	31	55	9.3	53	25	364	+	+	+
5	lSSc	ACA	29	55	54	18	68	37.2	962	22	1,180	+	+	+
6	dSSc	ATA PM-Scl	9.3	56	29	36	66	22.4	122	99	254	+	+	+
7	dSSc	ATA U3-RNP	16.2	54	14	21	68	17.8	109	52	271	+	+	+
8	lSSc	ACA	13.6	50	16	19	64	24.8	173	96	40	+	+	ND
9	dSSc	0	11.6	14	15	14	69	9.1	57	20	28	+	+	ND

+ means the presence of abnormalities described as criteria of myositis according to the EMNC (5); ACA, Anti-centromere antibodiy; ATA, Anti-topoisomerase I antibody; CK, creatine kinase; dSSc, diffuse form of systemic sclerosis; all patients had antinuclear antibody positive; EMG, electromyography; lSSc, limited cutaneous form of systemic sclerosis; ND, not done; PM-Scl, anti- polymyositis-scleroderma antibody; RMI, resonance magnetic imaging; U3-RNP, Anti U3-RNP antibody; Yrs, years

### Performances of muscular enzymes to predict subsequent myopathy related to SSc occurring three years after inclusion

The baseline plasma levels of aldolase ranged from 2 U/L to 40 U/L, those of CK from 22 U/L to 900 U/L, those of ALT from 6 U/L to 110 U/L and those of AST from 10 U/L to 60 U/L. The area under ROC curves (AUROCs) of aldolase and CK values for identifying subsequent Myo-SSc occurrence were 0.80 (95% CI: 0.67 to 0.94; *P *< 0.001), and 0.75 (95% CI: 0.49 to 1.0; *P *= 0.01), respectively. The AUROCs of ALT and AST values for identifying subsequent Myo-SSc occurrence were 0.63 (95% CI: 0.46 to 0.81; *P *= 0.04), and 0.57 (95% CI: 0.35 to 0.79; *P *= 0.14), respectively. The prediction power of plasma aldolase level for identifying subsequent Myo-SSc occurrence was significantly higher than those of CK, and those of transaminase enzymes (ALT, AST) but the difference between the two AUROCs was not statistically significant (*P *= 0.01). By contrast, CRP (AUROC = 0.48 for 95% CI: 0.28 to 0.70; *P *= 0.27) had no predictive value for Myo-SSc occurrence.

### Thresholds of muscular enzymes to predict subsequent myopathy related to SSc occurring three years after inclusion

The best cut-off of aldolase for prediction of subsequent Myo-SSc occurrence within three years after inclusion was 9 U/L (95% CI: 3.5 to 20.0) with a sensitivity of 89% (95% CI: 52% to 99%) and a specificity of 67% (58% to 75%). The best cut-off of CK, ALT and AST for identifying patients with high risk of developing Myo-SSc within three years after inclusion were 92 U/L (24.1 to 259.4), 18.0 U/L (9.7 to 32.7), and 25.0 U/L (13.2 to 39.2), respectively. These cut-offs were all below its upper limit of the normal level (the upper limit of the normal level of CK was 160 U/L, those of ALT and AST were 32 U/L and 32 U/L in females, 35 U/L and 43 U/L in males, respectively.

### Association between aldolase at baseline and subsequent Myo-SSc occurrence

**Figure 2:**
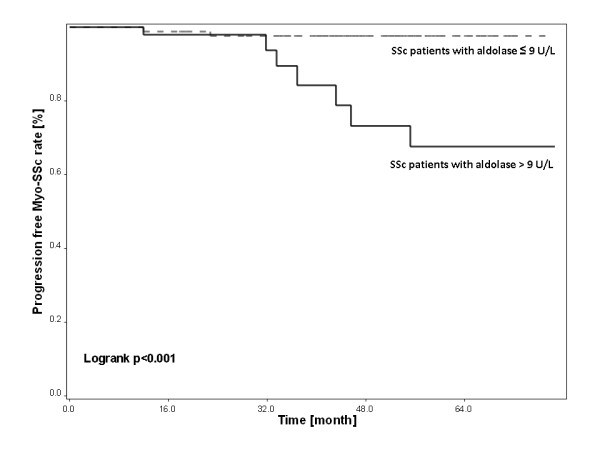
**Kaplan Meier analysis grouped by baseline aldolase**. The black line represents the group of SSc patients with baseline plasma aldolase level above 9 U/L and the dotted grey line the group of SSc patients with baseline plasma aldolase level equal or below 9 U/L.

Systemic sclerosis patients whose aldolase level at baseline was higher than 9 U/L were more likely to develop subsequent Myo-SSc within the three years of the course of disease (Figure 2). Clinical features and the presence of anti-PM-Scl antibody, anti-U1-RNP antibody, anti-U3-RNP antibody and anti RNA polymerase III antibody known as auto-antibodies associated with Myo-SSc, could not predict the subsequent Myo-SSc occurrence. In an unadjusted Cox model, aldolase had a predictive value on subsequent Myo-SSc occurrence (Hazard ratio (HR) = 11.1; 95% CI: 2.8 to 44.5, *P *< 0.001). In a Cox proportional hazards model adjusted for age, gender, and form and duration of disease, patients with aldolase higher than 9 UI/L had a higher risk of developing subsequent Myo-SSc occurrence (Hazard ratio (HR) = 10.3, 95% CI: 2.3 to 45.5; *P *< 0.001, Table 3).

**Table 3:** Risk subsequent muscular involvement according to aldolase at baseline after adjusting for other characteristics at baseline

	Hazard ratio (95% CI)	*P*
**Aldolase > 9 U/L**	10.3 (2.3 to 45.5)	< 0.001
**Age (per additional year)**	0.98 (0.93 to 1.04)	0.68
**Female**	0.63 (0.07 to 5.18)	0.67
**Diffuse form of disease**	1.23 (0.27 to 5.49)	0.78
**Duration of disease (per additional year)**	0.97 (0.90 to 1.05)	0.57

Upper limit of plama aldolase value: 7.0 UI/L

### Changes in muscular enzymes and CRP from baseline to the diagnosis of Myo-SSc

Plasma aldolase A from all patients remained higher than the upper limit of normality from inclusion to diagnosis of Myo-SSc and the trend was towards an increasing aldolase A level from baseline to the moment of Myo-SSc diagnosis except in two patients. AST and CK increased when the proximal strength weakness appeared. ALT levels were fluctuating between the inclusion and the moment of Myo-SSc diagnosis (Table 2).

### Influence of immunosuppressive therapy on the occurrence of Myo-SSc and its evolution

At inclusion, 18 SSc patients (13%) had immunosuppressive therapy. Six out of nine patients with Myo-SSc were treated with immunosuppressive agents before the diagnosis of Myo-SSc (Figure 1). At the time of final assessment, 40 patients (26.3%) out of 137 had immunosuppressive therapy (Methotrexate, cyclophosphamide, or mycophenolate mofetil) for recent lung function deterioration, or arthritis during the follow-up period. Among 22 patients who had newly immunosuppressive therapy during the follow-up, none had myositis.

All Myo-SSc patients had immunosuppressive therapy and intravenous immunoglobulin after diagnosis. Three out of nine normalized the 8-MMT score and aldolase levels. Although combining therapy, six remaining patients with myositis worsened, and three died due to acute respiratory failure (*n *= 2), and bacterial septicemia related to intestinal pseudo-obstruction with severe malnutrition (*n *= 1). Consecutive plasma aldolase levels from the six other Myo-SSc patients remained higher than the upper limit of normal value.

## Discussion

In this cohort study, we found that elevated plasma aldolase at inclusion was an accurate marker that helped to identify SSc patients without muscle weakness who were going to develop subsequent Myo-SSc during the follow-up period. Thus, the best threshold of aldolase for prediction of Myo-SSc occurrence within three years after inclusion was 9 U/L. As opposed to aldolase, elevated plasma CK or ALT had weaker performance for prediction of subsequent Myo-SSc occurrence in SSc, especially because their respective thresholds were below the upper limit of measurement method and made them unreliable to discriminate SSc patients with high risk from those with low risk. Moreover, AST and CRP were not able to predict subsequent Myo-SSc occurrence.

Clements *et al*. [16] suggested that there are two patterns of muscle involvement: on one hand, "simple myopathy", a non-progressive form of myopathy with normal or slight elevation of muscular enzymes characterized by muscle atrophy without inflammation; on the other hand, "scleromyositis", a progressive form with profound proximal muscle weakness, significant elevation of muscular enzymes, EMG abnormality and inflammatory myopathy in muscle biopsy specimens. Although these two patterns of myopathy are still relevant in the clinical setting, the design of our present study was aimed to diagnose the latter disabling form of My which needs corticosteroids and immunosuppressive therapy [3]. Our findings are consistent with the hypothesis that there is a gradual transition in the course of muscle involvement in SSc from early muscle damage related to inflammatory processes to the late stage of muscle atrophy and fibrosis leading to a proximal muscle weakness. As aldolase is a highly sensitive marker of muscle damage, the release of a high amount of this enzyme in blood plasma from SSc patients with normal muscle strength may reflect underlying muscle injury that herald severe Myo-SSc occurrence and may be used as an early sign of this disease.

Currently, patients with SSc have multiple complaints, including myalgia, skin hardening, joint pain or arthritis, that make the diagnosis of Myo-SSc more difficult and may even delay the diagnosis. To date, there is no relevant predictive marker available for Myo-SSc. Previous reports suggested that an increased aldolase level in patients with Myo-SSc reflect both the activity and severity of the myopathy [9,17,18]. Here, we provide clinical evidence that aldolase may be useful as a predictive marker of subsequent Myo-SSc occurrence in SSc patients without proximal muscle weakness. Indeed, patients with aldolase levels above 9 U/L were more likely to develop subsequent Myo-SSc. Hence, elevated aldolase in patients with SSc may be used by clinicians to monitor a close follow-up to choose the right time to perform invasive investigations, such as muscle biopsy, yielding critical information, allowing them to make early diagnosis of Myo-SSc and to give appropriate therapy [6]. Other muscular enzymes, including CK, ALT and AST, were not accurate in predicting the subsequent Myo-SSc occurrence.

The rate of anti-PM-Scl antibody found in this present study was consistent with that recently reported by Hanke *et al*. [19], but lower than that reported by others [20-22]. We did not find a discrepancy between immunodiffusion and immunodot methods for Pm-Scl antibody, suggesting indirectly the presence of isolated anti-PMScl-75 kDa antibody. However, the presence of antibodies against antitopoisomerase I, anti-centromere, anti-ribonucleoprotein or PM-Scl did not predict the subsequent Myo-SSc occurrence. This is at odds with the previous results, showing that patients with anti-PM-Scl antibody had pejorative prognosis in IIM [23]. The presence of anti-PM-Scl antibody could not estimate the risk of developing Myo-SSc in patients with SSc.

Proximal muscle weakness is a hallmark of myositis associated with connective tissue disease, and current available therapies attempt to restore muscle strength. Although the diagnosis criteria of Myo-SSc are not yet fully codified, they rely primarily on the proximal muscle weakness and require association with at least two of the three others criteria defined by the 119^th ^ENMC International Workshop [5]. Although muscle biopsy specimens from two patients with Myo-SSc were not available, all nine patients fulfilled myositis associated with connective tissue disease according to the 119^th ^ENMC International Workshop criteria [5].

One might argue that other factors, including physical activity and/or trauma, may contribute to elevation of plasma aldolase. In this study, aldolase was assessed in patients after 72 hours of resting, and overnight fasting. For obvious ethical reasons, we could not obtain muscle tissue to provide direct histological evidence showing myopathy or myositis in patients without proximal muscle weakness and isolated increase of aldolase. There is, however, circumstantial evidence, stemming from previous studies in SSc patients with Myo-SSc, that partly explain our findings. In particular, an autopsy study found inflammatory muscles in 9% of patients with SSc [24], with different patterns of myopathy; this suggests a continuum spectrum between the slight elevation of aldolase and "scleromyositis" during the course of disease.

Our study had several limits. First, consistently with a recent report [25], the incidence of Myo-SSc with proximal muscle weakness during a mean follow-up was low (6.6% in 45.3 months of follow-up), which may explain the wide range of hazard ratio. Second, although we provided accurate thresholds of aldolase for prediction of Myo-SSc occurrence, external validation by an independent cohort is needed prior to recommended intervention studies targeting aldolase in SSc. Third, we might neglect some early stage of myositis, but the annual visits scheduled and additional visits if symptoms occurred minimize the risk of misdiagnose mild myositis.

## Conclusion

Our findings show that increased plasma aldolase is a non-invasive and valuable means of identifying SSc patients with high risk of developing subsequent Myo-SSc. This would allow clinicians to monitor these at-risk patients and to diagnose Myo-SSc at an early stage. This would allow clinicians to initiate appropriate treatments when the muscle damage is still in a reversible stage.

## Abbreviations

8-MMT: eight manual muscle testing; ACA: anti-centromere antibody; ALT: alanine transaminase; ANA: antinuclear antibody; AST: aspartate transaminase; ATA: antitopoisomerase I antibody; AUROC: area under receiver operating characteristic curve; CI: confidence intervals; CK: creatine kinase; CRP: C-reactive protein; EMG: electromyography; ENMC: European Neuromuscular Centre; HR: hazard ratio; ID: immuno-diffusion; IIM: idiopathic inflammatory myopathy; Myo-SSc: myopathy related to systemic sclerosis; PM-Scl: anti-polymyositis-scleroderma antibody; RMI: resonance magnetic imaging; ROC: receiver operating characteristic curve; SD: standard deviation; SSc: systemic sclerosis; U1-RNP: anti-ribonucleoprotein antibody.

## Competing interests

The authors do not have a financial relationship with a commercial entity that has an interest in the subject of this manuscript.

## Authors' contributions

CT and KPT had full access to all of the data in the study and take responsibility for the integrity of the data and the accuracy of the data analysis. CT, MG, AK, BB, CJ, PC, SR, JC and KPT were responsible for study conception and design. CT, MG, AK, BB, CJ, PC, SR, JC and KPT were responsible for the acquisition of data. CT, MG, AK, BB, CJ, PC, SR, JC and KPT were responsible for the analysis and interpretation of data. All authors were involved in drafting the article or revising it critically for important intellectual content, and all authors approved the final version to be submitted for publication.
